# Reference evapotranspiration estimate with missing climatic data and multiple linear regression models

**DOI:** 10.7717/peerj.15252

**Published:** 2023-04-27

**Authors:** Deniz Levent Koç, Müge Erkan Can

**Affiliations:** Agricultural Structures and Irrigation/Agriculture Faculty, Çukurova University, Adana, Turkey

**Keywords:** Reference evapotranspiration, Missing climatic data, Multiple linear regression models, FAO-56 Penman-Monteith (PM)

## Abstract

The reference evapotranspiration (ETo) is considered one of the primary variables for water resource management, irrigation practices, agricultural and hydro-meteorological studies, and modeling different hydrological processes. Therefore, an accurate prediction of ETo is essential. A large number of empirical methods have been developed by numerous scientists and specialists worldwide to estimate ETo from different climatic variables. The FAO56 Penman-Monteith (PM) is the most accepted and accurate model to estimate ETo in various environments and climatic conditions. However, the FAO56-PM method requires radiation, air temperature, air humidity, and wind speed data. In this study in Adana Plain, which has a Mediterranean climate for the summer growing season, using 22-year daily climatic data, the performance of the FAO56-PM method was evaluated with different combinations of climatic variables when climatic data were missing. Additionally, the performances of Hargreaves-Samani (HS) and HS (A&G) equations were assessed, and multiple linear regression models (MLR) were developed using different combinations of climatic variables. The FAO56-PM method could accurately estimate daily ETo when wind speed (U) and relative humidity (RH) data were unavailable, using the procedures suggested by FAO56 Paper (RMSEs were smaller than 0.4 mm d^−1^, and percent relative errors (REs) were smaller than 9%). Hargreaves-Samani (A&G) and HS equations could not estimate daily ETo accurately according to the statistical indices (RMSEs = 0.772-0.957 mm d^−1^; REs (%) = 18.2–22.6; R^2^ = 0.604–0.686, respectively). On the other hand, MLR models’ performance varied according to a combination of different climatic variables. According to t-stat and p values of independent variables for MLR models, solar radiation (Rs) and sunshine hours (n) variables had more effect on estimating ETo than other variables. Therefore, the models that used Rs and n data estimated daily ETo more accurately than the others. RMSE values of the models that used Rs were between 0.288 to 0.529 mm d^−1^; RE(%) values were between 6.2%–11.5% in the validation process. RMSE values of the models that used n were between 0.457 to 0.750 mm d^−1^; RE(%) values were between 9.9%–16.3% in the validation process. The models based only on air temperature had the worst performance (RMSE = 1.117 mm d^−1^; RE(%) = 24.2; R^2^ = 0.423).

## Introduction

Evapotranspiration plays a crucial role in the hydrological cycle and is a significant cause of worldwide water loss. Almost 62% of the precipitation that falls on continents returns to the atmosphere through evapotranspiration (Dingman, 2002). Therefore, accurate evaluation of evapotranspiration is essential from various aspects: irrigation scheduling, water resource management, irrigation system design and management, crop yield simulation, hydrological design, and accurate quantification of hydrological water balance (Izadifar, 2010).

ETo can be multiplied by a crop-specific coefficient for estimating crop evapotranspiration (ETc) (Allen et al., 1998). Many studies worldwide have shown the FAO56-PM equation to be the most accurate ETo method under various climatic conditions (Allen et al., 1998; Todorović, Karic & Pereira, 2013; Kisi, 2016). However, the main restriction of using this method is that it requires weather data, some of which can only be acquired from major weather stations. The absence of these measurements in rural areas, especially in developing countries, limits the usability of the FAO56-PM equation. However, this has encouraged studies in estimating ETo values from easily obtainable climatic measurements, such as temperature and wind speed (Zouzou & Citakoglu, 2021).

Many empirical and semi-empirical equations have been developed to estimate ETo, which most require one or more weather data sets (Turc, 1961; Snyder, 1992; Hargreaves & Samani, 1985). Since the performances of these equations in different environments are varied, FAO has suggested using missing climatic data procedures in the FAO56-PM method instead of these equations (Allen et al., 1998).

Many publications have referred to testing alternative equations with FAO56-PM ETo calculated with complete weather data sets (Sentelhas, Gillespie & Santos, 2010; Todorović, Karic & Pereira, 2013). Under climatic data-limited conditions, the Hargreaves–Samani (HS) equation which requires air temperature only, has frequently been used to estimate ETo (Hargreaves & Samani, 1985). Another approach suggested by Allen et al. (1998) is to use only a set of temperature data, often referred to in the literature as a reduced set FAO56-PM equation (FAO56-PMT).

Several studies have evaluated the accuracy of the FAO56-PM equation using only maximum and minimum temperature data (FAO56-PMT) by comparing it with results of FAO56-PM, which has complete data and other ETo equations, mainly HS (Stockle, Kjelgaard & Bellocchi, 2004; Popova, Kercheva & Pereira, 2006; Jabloun & Sahli, 2008; Todorović, Karic & Pereira, 2013).

Stockle, Kjelgaard & Bellocchi (2004) evaluated the accuracy of the FAO56-PM method in five locations in the Netherlands, Spain, the Philippines, the USA, and Syria using the procedure suggested by FAO56 by estimating Rs and vapor-pressure deficit from temperature data. They found that the daily estimate of Rs and VPD for arid and semi-arid locations changed between marginal to acceptable and were poor for other areas. Also, the estimated FAO56-PM ETo appeared suitable for the arid and semi-arid areas weekly, whereas daily ETo estimations were generally poor in all locations.

According to a study performed by Popova, Kercheva & Pereira (2006), when solar radiation data were not observed, the procedures suggested by the FAO56 Paper to estimate Rs from Tmax and Tmin gave accurate estimates of ETo. When air humidity data were missing, replacing Tdew (daily dewpoint temperature) with Tmin for computing the actual vapor pressure (e_a_) was appropriate. When U data were unavailable, in case the regional average wind speed was used instead of actual wind speed, accurate ETo estimates were obtained. When only temperature data were observed, FAO-56 PMT provided better results than HS.

Jabloun & Sahli (2008) reported similar results for different places in Tunisia. When Rs data were missing, the Rs procedure using air temperature differences given by FAO56 Paper yielded accurate ETo estimates. Using Tdew = Tmin in the FAO56-PM model was a good alternative to estimate e_a_ when measured RH data were missed. Using the regional average wind speed instead of daily data gave accurate estimates of ETo, and the FAO56-PMT gave more precise results than HS in the study.

Under a monsoon climate, an application to the North China Plain has shown that FAO56-PMT daily estimates fit the FAO56-PM ETo estimates better and produced more minor estimation errors than HS (Liu & Pereira, 2001; Pereira, Cai & Hann, 2003).

Annandale et al. (2002) successfully applied FAO56-PMT to varied climates in South Africa and suggested using 5-day average ETo values instead of daily ETo values.

In Southern Ontario, Sentelhas, Gillespie & Santos (2010) investigated 13 alternatives to estimate ETo with different availabilities of climate data. When wind speed (U) and relative humidity (RH) data were unavailable for the FAO56-PM method to calculate ETo, the following cases gave accurate results: using mean wind speed data in a close area within the same homogenous region instead of actual wind speed data; replacing Tdew with Tmin for to be computing the actual vapor pressure.

Benli et al. (2010) evaluated the performance of FAO56-PM in case of missing climatic parameters in a semi-arid highland environment in Turkey. When wind speeds were not observed, an average wind speed of 2 m/s for light to moderate wind gave acceptable results to estimate ETo in this environment. In addition, when air humidity data were missing, in computing actual vapor pressure (e_a_), using Tmin instead of Tdew indicated acceptable results to estimate ETo.

When climatic data are missing, the multiple linear regression (MLR) approaches are also used to estimate ETo. Standard MLR is a known statistical modeling technique for data mining and function estimation problems among the data-driven modeling approaches. Despite the considerable development in data-driven modeling, MLR is still popular and used for various modeling and model comparison issues due to its simplicity. MLR approach has been used in a few studies in different parts of the world for modeling the ETo (Irmak et al., 2003; Dai et al., 2008; Izadifar, 2010; Choi et al., 2018; Mattar & Alazba, 2019; Yirga, 2019; Mohsin & Lone, 2021; Kim, Bae & Jang, 2022; Dimitriadou & Nikolakopoulos, 2022). Results have shown that obtained models by the MLR approach estimated ETo successfully.

Irmak et al. (2003) modeled ETo by MLR technique in North-Central Florida, which has a humid climate using 21 years of daily climate data. Their study derived two models based on the first Rs, Tmax, and Tmin, and the second Rn, Tmax, and Tmin. The study used the fifteen-year dataset (1980–1994) to calibrate the model parameters. The six-year dataset (1995–2000) was used to validate the models. The models’ daily, weekly, and annual total ETo estimations were very close to those obtained from the FAO56-PM method.

Dai et al. (2008) estimated ETo using MLR and artificial neural network (ANN) models with four and three inputs in China’s arid, semi-arid, and sub-humid areas. Both four and three-input MLR and ANN models gave similar results for the sub-humid regions. In comparison, ANN models outperformed MLR models for arid and semi-arid areas.

Izadifar (2010) investigated data-driven modeling techniques for estimating the hourly actual evapotranspiration mechanism. Genetic programming (GP) and MLR data-driven modeling techniques performed similarly and better than the ANN model concerning the ability to generalize.

Choi et al. (2018) tested the neural network models and MLR algorithms. They reported that the performance of the MLR model was not inferior as long as there was no derivation of negative values.

Mattar & Alazba (2019) used MLR models and gene expression programming (GEP) to estimate the mean monthly ETo with limited climatic data in Egypt. In the study, MLR model ETo estimates indicated the same results trend with GEP models in the training and testing subset.

Yirga (2019) modeled ETo by MLR approach for the Megecha catchment. The results showed that the model with five variables (U, Tmax, Tmin, N, RH) estimated monthly ETo with excellent performance.

Mohsin & Lone (2021) developed models using by MLR approach to estimate ETo for Kashmir Valley. R^2^ values for the developed models were greater than 0.96.

Kim, Bae & Jang (2022) used MLR and PR algorithms to estimate ETo under climatic data-limited conditions with data obtained from 62 climate stations across South Korea. In the study, the best R^2^ were as follows: 0.62, 0.69 (daily), 0.77, 0.81 (monthly), and 0.40, 0.49 (annual) for MLR and PR algorithms, respectively. Models applicability was different inland and coastal areas. So it was recommended to apply the most suitable model for each region by examining other training data and learning models, considering regional characteristics in the study.

Dimitriadou & Nikolakopoulos (2022) investigated the utility of MLR in estimating ETo in the Peloponnese-Greece, for two typical winter and summer months during 2016–2019. The results of the study indicated that MLR could produce outstanding predictive models.

The procedures suggested by Allen et al. (1998) for predicting missing climatic parameters require assessment in different environments and climates to test their applicability because the results vary according to climatic conditions (Valle Júnior et al., 2021). No studies have addressed assessing the performance of FAO56-PM with missing climatic data to estimate daily ETo in this region of Turkey. In this study, MLR models were also used to estimate ETo because the added value of MLR models is the simplicity and comprehensibility of the method and the equations produced. MLR models give us the formula or relationship and have acceptable accuracy. The advantage of this method is the easy interpretation of the coefficients generated in the model with low computational effort compared to more complex techniques, such as artificial intelligence algorithms (Dimitriadou & Nikolakopoulos, 2022). No previous study modeled ETo using daily climate data by MLR approach in this region of Turkey. So, this study intends to close these gaps in the literature. Also, daily ETo estimation instead of monthly ETo estimation would help professionals in real-time irrigation scheduling, where daily ETo values are needed.

According to this, the specific objectives of this study are:

(1) To assess the performance of the FAO56-PM method using the missing climatic data procedure with different combinations of climatic variables to estimate daily ETo.

(2) To assess the performance of HS and HS (A&G) equations to estimate daily ETo.

(3) To develop MLR models using available climatic variables and compare their performance with the FAO56-PM method with full data to estimate the daily ETo.

(4) To identify the most critical climatic variables affecting the ETo process and to identify, using the statistical criteria, the supremacy of one modeling approach over the other models.

## Materials & Methods

### Study area

The Adana Plain is located in the Mediterranean Region and consists of two parts, Çukurova Plain and Upper Plain. This study selected the Adana plain because It is Turkey’s most extensive and fertile delta plain. Moreover, several crops can be planted in the same crop year in Adana, where fertile cultivated land occupies a large area due to the suitable climate. Cereal, fruit, vegetable, and citrus are grown in the region, and their production efficiency is high (KafalıYılmaz, 2019). In Turkey, Adana Plain ranks first in citrus, watermelon, soybean, and peanut production (Kadeş, 2019). Rainfall amounts and distribution are insufficient during the growing season, so irrigation is indispensable in crop production in Adana Plain. This study includes April to October, covering the growing season of the main crops in the Adana Plain. Long-dated daily climate variables covering the period from 1998 to 2019 were used in the study.

**Figure 1:**
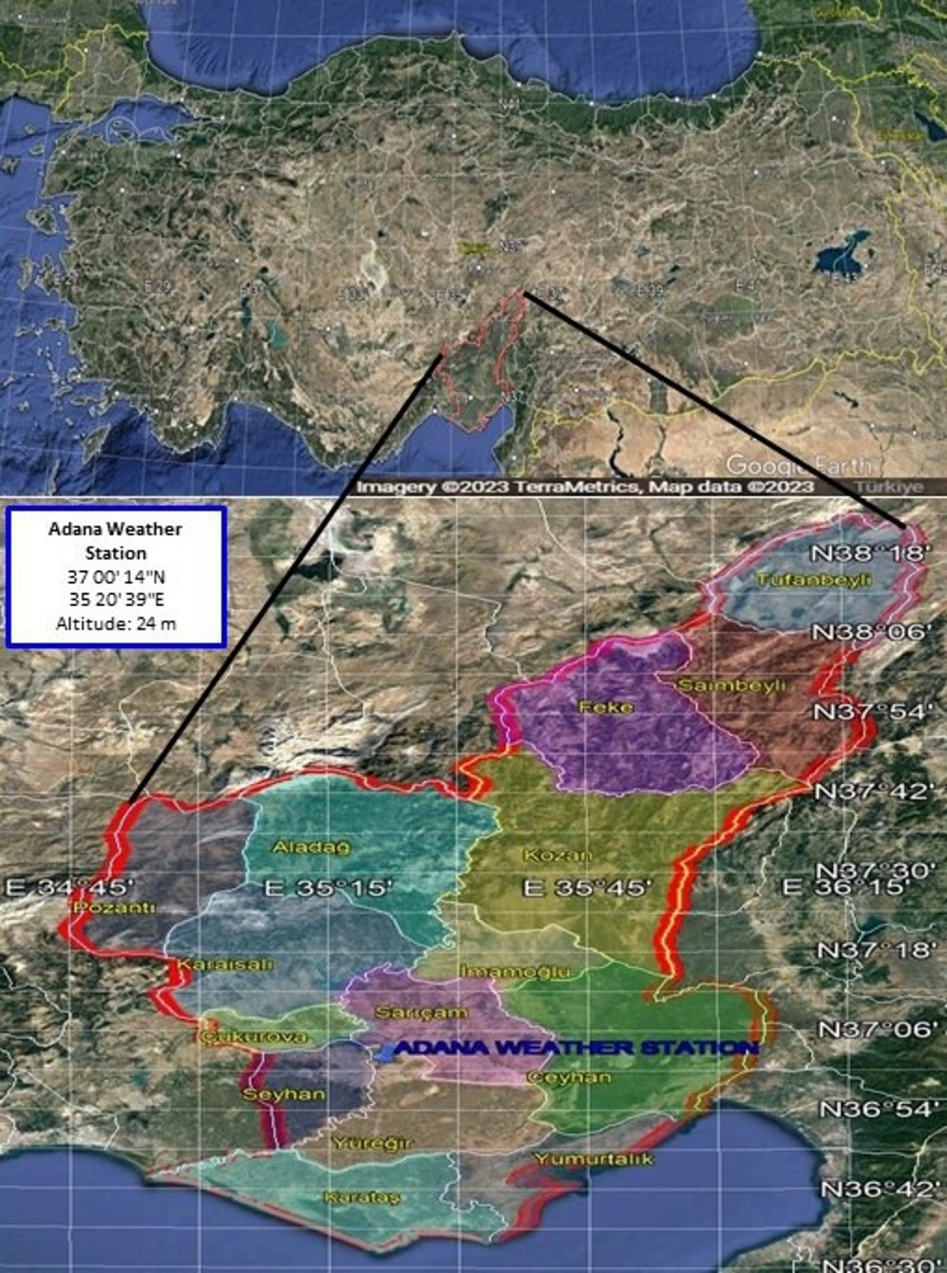
Location of the Adana weather station in Adana Plain in Turkey. Map data: Google, CNES/Airbus, Landsat/Copernicus, Maxar Technologies, 2023.

This study used the Adana weather station in the center of Adana, operated by the Turkish State Meteorological Service (TSMS) (Fig. 1). Adana has a hot-summer Mediterranean climate. According to the Köppen-Geiger classification, Adana has a warm temperate climate with dry summer and hot summer (classified as Csa). (Öztürk, Çetinkaya & Aydın, 2017). The annual average (1929-2021) precipitation is 668.7 mm, of which about 50% of rainfall occurs in December, January, and February. The yearly average (1929–2021) sunshine hours and temperature are 7.5 h and 19.2 °C, respectively (Turkish State Meteorological Service, 2022).

### FAO56-Penman-Monteith (PM) method

FAO recommends the FAO56-PM method as the sole standard approach to estimating ETo and validating other models, and It is expressed as (Allen et al., 1998):

(1) \begin{eqnarray*}ETo= \frac{0.408\times \Delta \times \left( {\mathrm{R}}_{\mathrm{n}}-\mathrm{G} \right) +\gamma \times \frac{900}{{{T}_{m}}_{c}+273} \times {\mathrm{U}}_{2}\times ({\mathrm{e}}_{\mathrm{s}}-{\mathrm{e}}_{\mathrm{a}})}{\Delta +\gamma \times (1+0.34\times {\mathrm{U}}_{2})} \end{eqnarray*}

(2) \begin{eqnarray*}& & {e}_{\mathrm{s}}= \frac{0.6108\times exp \left[ \frac{17.27\times Tma{x}_{c}}{Tma{x}_{c}+237.3} \right] +0.6108\times exp \left[ \frac{17.27\times Tmi{n}_{c}}{Tmi{n}_{c}+237.3} \right] }{2} \end{eqnarray*}

(3) \begin{eqnarray*}& & {e}_{\mathrm{a}}= \frac{RH}{100} \times {e}_{\mathrm{s}}\end{eqnarray*}

(4) \begin{eqnarray*}\Delta = \frac{4098\times \left[ 0.6108\times \exp \nolimits ( \frac{17.27\times {{T}_{m}}_{c}}{{{T}_{m}}_{c}+237.3} ) \right] }{({{T}_{m}}_{c}+237.3)^{2}} \end{eqnarray*}

The wind speed data measured at 10 m was converted to a standard height of 2 m using Eq. (5) (Allen et al., 1998) (Eq. (5)).

(5) \begin{eqnarray*}{U}_{2}={U}_{Z}\times \left[ \frac{4.87}{\ln \nolimits (67.8\times Z-5.42)} \right] .\end{eqnarray*}

Where ETo = reference evapotranspiration (mm d^−1^); R_n_ = net radiation (MJ m^−2^ d^−1^); G = soil heat flux density (MJ m^−2^ d^−1^), considered zero for daily estimates; Δ = slope of vapor pressure curve (kPa. °C^−1^); *γ* = psychrometric constant (0.0672 kPa K^−1^); Tm_c_ = mean air temperature (°C); *Tmax*_*C*_ = maximum air temperature (°C); *Tmin*_*C*_ = minimum air temperature (°C); U_2_ = wind speed at 2 m height (m s^−1^); Z = height of the wind speed measurement (10 m); *e*_s_ = saturation vapor pressure (kPa); e_a_ = actual vapor pressure (kPa); (e_s_ − *e*_a_) = saturation vapor pressure deficit (kPa); RH = mean relative humidity (%).

(6) \begin{eqnarray*}& & Rn=Rns-Rnl\end{eqnarray*}

(7) \begin{eqnarray*}& & Rns=0.77\times Rs\end{eqnarray*}

(8) \begin{eqnarray*}& & Rnl= \left[ \sigma \times \right. \left( \frac{Tma{x}_{K}^{4}+Tmi{n}_{K}^{4}}{2} \right) \times \left( 0.34-0.14\times \sqrt{{e}_{a}} \right) \times \left( 1.35\times \frac{{R}_{s}}{{R}_{so}} -0.35 \right) \end{eqnarray*}

(9) \begin{eqnarray*}& & Rso=0.75xRa\end{eqnarray*}

where Rns = net solar radiation (MJ m^−2^ d^−1^); Rnl = net longwave radiation (MJ m^−2^ d^−1^); Rs = solar radiation (MJ m^−2^ d^−1^); Rso = clear-sky solar radiation; Ra = extraterrestrial radiation (MJ m^−2^ d^−1^); *Tmax*_*k*_ = maximum absolute temperature (K); *Tmin*_*k*_ = minimum absolute temperature (K).

Daily values of Ra were computed using the equations given in the FAO56 Paper by Allen et al. (1998).

### Procedures for estimating missing climatic data

The FAO56-PM method can only be used when a full climatic dataset is available. However, Allen et al. (1998) suggested the following procedures for estimating absent Rs, U, Δe data:

### Estimating missing solar radiation (Rs) data with the Angstrom formula

Rs is often estimated from sunshine data using the Angstrom formula (Eq. (10))

(10) \begin{eqnarray*}{R}_{s}= \left( {a}_{s}+{b}_{s}\times \frac{n}{N} \right) \times {R}_{a}\end{eqnarray*}

where N = daylight hours (hour); n = actual sunshine duration (hour). a_s_ = 0.25; b_s_ = 0.50 are recommended where no actual Rs data are available (Allen et al., 1998). This study used as a_s_ = 0.25 and b_s_ = 0.50. In this study, Rs estimated from sunshine data using the Angstrom formula is denoted by the symbol Rs_n_.

### Estimating missing solar radiation (Rs) data with the Hargreaves’ radiation formula

Where sunshine data are lacking, the Hargreaves’ radiation formula can be used to estimate Rs. (Eq. (11)) (Allen et al., 1998).

(11) \begin{eqnarray*}{R}_{s}={K}_{rs}\times \sqrt{ \left( Tma{x}_{C}-Tmi{n}_{c} \right) }\times {R}_{a}\end{eqnarray*}

Where Krs = adjustment coefficient (≅0.16 for inland areas dominated by landmass; ≅ 0.19 for coastal areas adjacent to the coast of a large land mass). This study used 0.16 for Krs. In this study, Rs estimated from air temperature differences using the Hargreaves’ radiation formula is denoted by the symbol Rs_T_.

### Procedure for estimating wind speed (U)

Where wind speed data are not measured, mean wind speed data observed at a close location within the same homogenous region can be used instead of actual wind speed data, as proposed by Allen et al. (1998). This study used the closest weather station’s average wind speed (U_2_) of 0.95 m s^−1^ (1998–2019) instead of observed data.

### Procedure for estimating vapor pressure deficit (es − ea =Δe)

Where air humidity data are missing, vapor pressure deficit ( Δe) can be calculated based on temperature data suggested by Allen et al. (1998). *e*_a_ is obtained by assuming that the dew point temperature (Tdew) is close to the daily minimum temperature (Tmin). Then, *e*_a_ iscalculated by Eq. (12).

(12) \begin{eqnarray*}{e}_{a}=0.6108\times \exp \nolimits ( \frac{17.27\times Tmi{n}_{c}}{Tmi{n}_{c}+237.3} )\end{eqnarray*}

### Hargreaves-Samani (HS) and HS (A & G) equations

FAO suggests that when Rs, RH, and U data are missing, ETo can be estimated with the Hargreaves-Samani equation only using Tmax and Tmin (Hargreaves & Samani, 1985) and is expressed by Eq. (13):

(13) \begin{eqnarray*}ETo=0.0023\times Ra\times { \left( Tma{x}_{c}-Tmi{n}_{c} \right) }^{0.5}\times ({{T}_{m}}_{c}+17.8).\end{eqnarray*}

In this study, a modified HS equation developed by Almorox & Grieser (2016) for a warm temperate climate with dry summer (as classified Cs) was also used to estimate ETo (Eq. (14)).

(14) \begin{eqnarray*}ETo=0.00419\times Ra\times { \left( Tma{x}_{c}-Tmi{n}_{c} \right) }^{0.2342}\times \left( {{T}_{m}}_{c}+17.8 \right) -0.0208.\end{eqnarray*}

### Multiple linear regression (MLR) models

MLR is a statistical model that includes multiple predictive variables. The general form of the MLR model is:

(15) \begin{eqnarray*}Y={\beta }_{0}+{\beta }_{1}{X}_{1}+{\beta }_{2}{X}_{2}+\ldots +{\beta }_{n}{X}_{n}\end{eqnarray*}

where Y is the dependent variable; X_1_, …, and X_n_ are independent variables; ß_0_ is the unknown intercept; ß_1_, …, ß_n_ are partial regression coefficients of the function.

Multiple regression analysis was performed to develop linear models to estimate daily ETo values from meteorological variables, using ETo results based on calculations made by the FAO-56 PM equation (Tmax, Tmin, RH, U, Rs). The FAO56-PM ETo was employed as the dependent variable, while meteorological parameters were used as independent variables. The t-stat and *p*-values were examined to determine whether the best-fit line adequately fits the data. In several previous studies (Irmak et al., 2003; Noi, Degener & Kappas, 2017; Mattar & Alazba, 2019), one of the most common validation methods is dividing sample data into calibration and a validation dataset (for example, 70% and 30%, respectively). This study developed the model parameters using fifteen years (70%) of daily climatic data (April 1, 1998–October 31, 2012). Seven years (30%) of daily climatic data (April 1, 2013–October 31, 2019) were used to validation of the models.

### Data analysis

The ETo estimated using the MLR models and FAO56-PM with missing data were compared with the ETo data computed using the FAO56-PM equation with complete datasets. The performance of the methods and models was determined by regression analysis. The performances of ETo estimates were also evaluated using the following equations below, suggested by Willmott (1981), Karunanithi et al. (1994), and Jacovides & Kontoyiannis (1995). All the statistical indices were calculated daily.

(16) \begin{eqnarray*}& & d=1- \left[ \frac{\sum _{i=1}^{n}({P}_{i-}{O}_{i})^{2}}{\sum _{i=1}^{n}( \left\vert {P}_{i}-\overline{O} \right\vert + \left\vert {O}_{i}-\overline{O} \right\vert )^{2}} \right] ,(0\leq d\leq 1)\end{eqnarray*}

(17) \begin{eqnarray*}& & RMSE=\sqrt{ \frac{1}{n} \sum _{i=1}^{n}({P}_{i}-{O}_{i})^{2}}\end{eqnarray*}

(18) \begin{eqnarray*}& & RE= \frac{RMSE}{\overline{O}} \end{eqnarray*}

(19) \begin{eqnarray*}& & MBE= \frac{1}{n} \sum _{i=1}^{n}({P}_{i}-{O}_{i})\end{eqnarray*}

(20) \begin{eqnarray*}& & MAE= \frac{1}{n} \sum _{i=1}^{n} \left\vert {P}_{i}-{O}_{i} \right\vert \end{eqnarray*}

where n = the total number of data; P_i_ = estimated ETo values (mm d^−1^); O_i_ = FAO56-PM ETo values (mm d^−1^); $\overline{O}$ = the mean of FAO56-PM ETo values (mm d^−1^); d = the index of agreement; RMSE = root mean square errror; RE = relative error; MBE = mean bias error; MAE = mean absolute error.

In this study, the criteria given below, suggested by Stockle, Kjelgaard & Bellocchi (2004), were used to interpret the performance statistics of the methods in conjunction with the RMSE, MAE, MBE, b (slope), and a (intercept). The score is very good if *d* ≥ 0.95 and RE ≤ 0.10. The score is good if *d* ≥ 0.95 and 0.10 <RE ≤ 0.15. The score is acceptable if *d* ≥ 0.95 and 0.15 <RE ≤ 0.20. The score is marginal if *d* ≥ 0.95 and 0.20 <RE ≤ 0.25. Other combinations of d and RE values and R^2^ <0.85 indicate poor performance.

## Results & Discussion

### Estimating daily ETo with missing data, using the FAO56-PM method, HS and HS(A&G) equations

**Figure 2:**
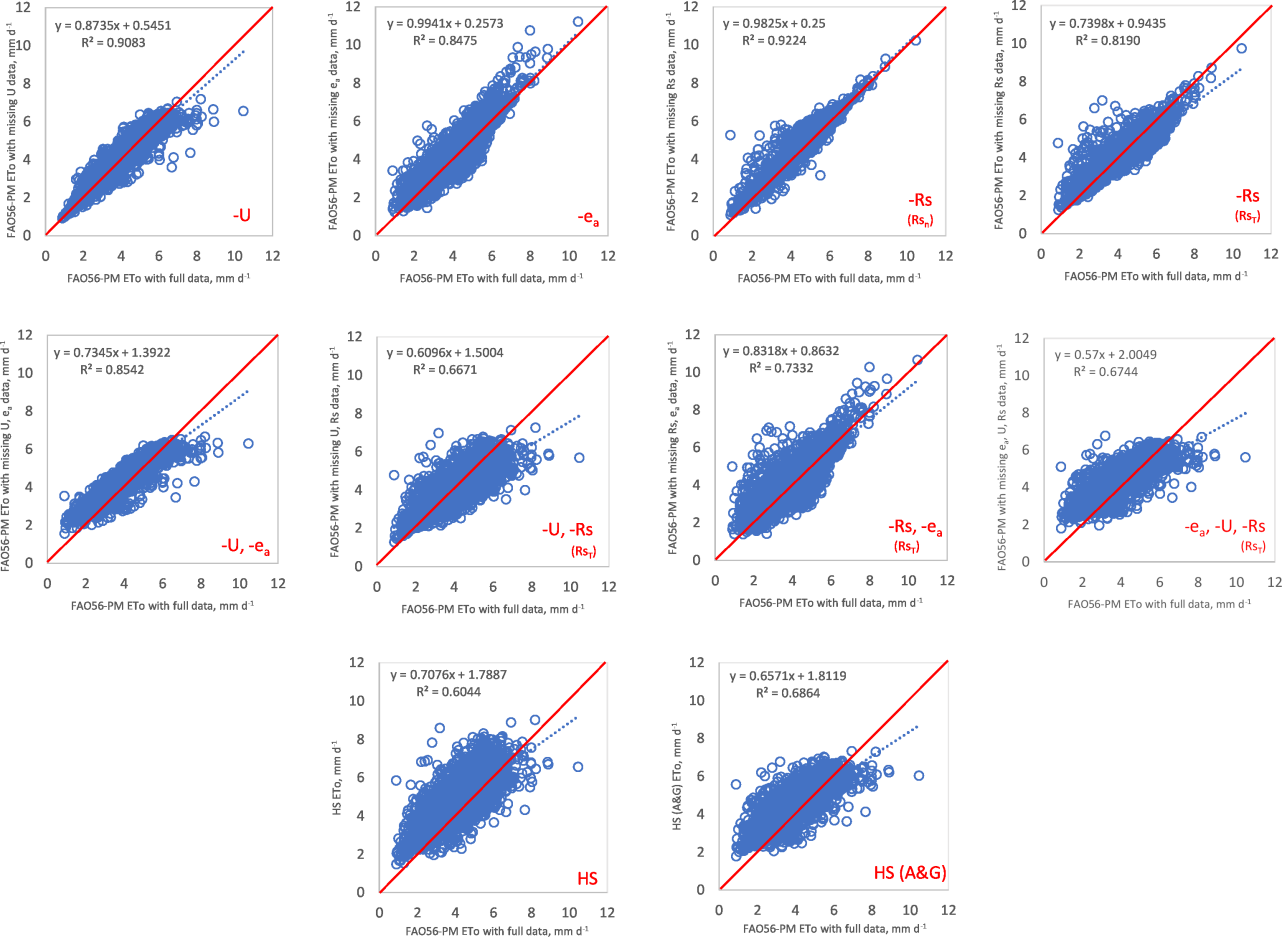
Scatter plots between calculated FAO56-PM ETo with complete dataset and estimated FAO56-PM ETo with missing data (*N* = 4,708 days).

The relationships between the ETo values estimated by the FAO56-PM method with complete data and those with the FAO56-PM method with missing data, HS, HS(A&G) equations are given in Fig. 2. It can be seen from Fig. 2 that the FAO56-PM ETo with missing U data correlated well with the FAO56-PM method with complete climatic data. Concerning regression equations, FAO56-PM with missing Rs data (Rs_n_) gave the best-predicted values: a slope close to unity (0.9825) and intercept close to zero (0.25), and coefficient of determination close to one (*R*^2^ = 0.9224). The FAO56-PM with missing *e*_a_ data gave the second-best values: (*b* = 0.9941, *a* = 0.2573, and R^2^ = 0.8475). It is clear from Fig. 2 that all methods underestimated and overestimated ETo values during the growing season. However, the FAO56-PMT method using only a set of temperature data referred to in the literature as a reduced set FAO56-PM equation generally overestimated ETo for more days. HS(A&G) equations gave similar results, as well. The FAO56-PMT method overestimated the ETo during 2,907 days and underestimated the ETo during 1,801 days. These overestimates and underestimates were 3,696 days and 1,012 days for the HS equation, whereas 3,356 and 1,352 days for HS(A&G) equation, respectively.

Table 1 shows the performance statistics between ETo calculated by the FAO56-PM with a complete dataset and estimated by the FAO56-PM with missing data and estimated by the HS and HS(A&G) equations. The daily methods’ ranking was made, considering all statistical indices in Table 1. As shown in Table 1, when U data were missing, the closest weather station’s average wind speed of 0.95 m s^−1^ was a perfect option for the FAO56-PM method because of minor errors. This method’s score was accepted as very good according to the criteria given by Stockle, Kjelgaard & Bellocchi (2004), and had the lowest RMSE, MAE, RE, and MBE values among the methods and ranked first (RMSE = 0.372 mm d^−1^, MAE = 0.248 mm d^−1^, RE = 0.088, and MBE = 0.009 mm d^−1^). Sentelhas, Gillespie & Santos (2010) found similar results in Southern Ontario, Canada. When just U data were missing, the standard U data from a nearby weather station was a perfect option for the FAO56-PM method due to minor errors. Allen et al. (1998) also suggest using a wind speed value of 2 m s^−1^ when wind speed data are unavailable. However, in our research, 98% of data from measurements showed U values below 2 m s^−1^. The studies by Popova, Kercheva & Pereira (2006) in South Bulgaria and Córdova et al. (2015) in the high Andes of southern Ecuador presented that the results of RMSE and MBE were near zero when using a wind speed value of 2 m.s^−1^. Djaman et al. (2016) reported that the performance of FAO56-PM was unsuitable in dry conditions when U was considered as 2 m s^−1^. However, when using the daily average U in the same conditions, FAO56-PM estimated ETo accurately with MBE values between −0.05 to 0.04.

**Table 1:** Statistical performances of daily ETo estimating methods tested against the FAO56-PM equation with full data.

**Methods**	**FAO56-PM method with missing data**							**FAO56-PMT**	**HS** **(A&G)**	**HS**
Missing data	-U	-Rs Rs(n)	-e_a_	-Rs Rs(T)	-U, -e_a_	-e_a_, -Rs Rs(T)	-U, -Rs Rs(T)	-e_a_, -U, -Rs Rs(T)	–	–
Parameters Used	Tmax, Tmin, e_a_, e_s_, Rs, Ra	Tmax, Tmin, e_a_, e_s_, U, Ra, n	Tmax, Tmin, e _s_, Rs, Ra, U	Tmax, Tmin, e _a_, e _s_, U, Ra	Tmax, Tmin, Rs, e _s_, Ra	Tmax, Tmin, e _s_, Ra, U	Tmax, Tmin, e _a_, e _s_, Ra	Tmax, Tmin, e _s_, Ra	Tmax, Tmin, Ra	Tmax, Tmin, Ra

**Ranking**	1	2	3	4	5	6	7	8	9	10
d	0.974 *(2)	0.975 *(1)	0.950 *(3)	0.936 *(4)	0.934 *(5)	0.921 *(6)	0.877 *(7)	0.866 *(9)	0.872 *(8)	0.830 *(10)
R^2^	0.908 *(2)	0.922 *(1)	0.850 *(4)	0.819 *(5)	0.854 *(3)	0.733 *(6)	0.667 *(7)	0.674 *(9)	0.686 *(8)	0.604 *(10)
RMSE (mm d^−1^)	0.372 *(1)	0.390 *(2)	0.564 *(5)	0.552 *(3)	0.559 *(4)	0.662 *(6)	0.724 *(7)	0.735 *(8)	0.772 *(9)	0.957 *(10)
RE	0.088 *(1)	0.092 *(2)	0.133 *(5)	0.130 *(3)	0.132 *(4)	0.156 *(6)	0.171 *(7)	0.174 *(8)	0.182 *(9)	0.226 *(10)
MAE (mm d^−1^)	0.248 *(1)	0.272 *(2)	0.421 *(4)	0.414 *(3)	0.447 *(5)	0.462 *(6)	0.558 *(7)	0.566 *(8)	0.595 *(9)	0.750 *(10)
MBE (mm d^−1^)	0.009 *(1)	0.176 *(5)	0.232 *(7)	−0.158 *(4)	0.268 *(8)	0.151 *(2)	−0.153 *(3)	0.184 *(6)	0.360 *(9)	0.550 *(10)
b (slope)	0.8735 *(3)	0.9825 *(2)	0.9941 *(1)	0.7398 *(5)	0.7345 *(6)	0.8318 *(4)	0.6096 *(9)	0.5700 *(10)	0.6571 *(8)	0.7076 *(7)
a (intercept)	0.5451 *(3)	0.2500 *(1)	0.2573 *(2)	0.9435 *(5)	1.3922 *(6)	0.8632 *(4)	1.5004 *(7)	2.0049 *(10)	1.8119 *(9)	1.7887 *(8)

**Notes.**

HS (A&G) A modified HS equation developed by Almorox & Grieser (2016).

An asterisk (*) indicates daily estimation rank number for each statistical indices.

When e_a_, usually obtained from relative humidity, was missing; in the case of estimating *e*_a_ by assuming that dew point temperature (Tdew) is close to the daily minimum temperature (Tmin), the agreement between ETo calculated with the complete data and ETo estimated with missing data indicated good performance due to low errors (RMSE = 0.564 mm d^−1^, MAE = 0.421 mm d^−1^, RE = 0.133, and MBE = 0.232 mm d^−1^). Several locations presented similar results when e_a_ was estimated by Tmin (Jabloun & Sahli, 2008; Popova, Kercheva & Pereira, 2006; Djaman et al., 2016). The study by Jabloun & Sahli (2008) obtained good results for sub-humid and semi-arid locations of Tunisia when e_a_ was estimated by Tmin. Excluding Tunis, for all other locations, the RMSE and MBE values ranged from 0.233 to 0.303 mm d^−1^ and from −0.212 to −0.078 mm d^−1^. In the study performed by Popova, Kercheva & Pereira (2006) in the Trace plain of south Bulgaria, when e_a_ was estimated by Tmin, regression coefficients (R^2^) were found close to 1, the slope of simple linear regression (b) ranged from 0.93 to 0.97, and the values of the standard error of estimates (SEE) ranged from 0.15 to 0.27 mm d^−1^. Similarly, Djaman et al. (2016) obtained good results in different agro-ecological zones of Burkina Faso. In the study, the values of b varied from 0.90 to 1.14, R^2^ ranged from 0.77 to 0.92, and RMSE and MBE varied from 0.07 to 0.22 mm d^−1^ and from −0.45 to 0.22 mm d^−1^, respectively.

When Rs data were missing, using the Angstrom formula to estimate Rs was a perfect option, with very low errors (RMSE = 0.390 mm d^−1^, MAE = 0.272 mm d^−1^, RE = 0.092, and MBE = 0.176 mm d^−1^). Its score can be accepted as very good. If sunshine hours were not measured, using Hargreaves’ radiation formula to estimate Rs was unacceptable due to *d* < 0.95 and R^2^ <0.85. So, Its score can be accepted as poor. In addition, the slopes of regression and intercept between ETo calculated with the complete data and those estimated with missing Rs data, *b* = 0.7398 and *a* = 0.9435, are not good values. However, when Hargreaves’ radiation formula calculates the Rs (Rs_T_), the ETo estimates were more accurate than the FAO56-PMT method, HS, and HS(A&G) equations according to all statistical indices (Table 1). Different studies using sunshine and temperature-based models to estimate Rs observed similar results to our research. The study by Jahani, Dinpashoh & Nafchi (2017) in Iran showed that sunshine-based models had more accurate estimations than temperature-based ones. The study by Trnka et al. (2005) in Central European lowlands presented that the Angstrom-Prescott model was the best method for estimating Rs. Aladenola & Madramootoo (2014) recommended the Hargreaves’ radiation formula for estimating Rs in Canada’s absence of solar radiation and sunshine data, unlike our research. More research is needed to find the best model for estimating Rs for different locations.

As seen in Table 1, for conditions in which U and e_a_ data were missing (-U, -e_a_), the ETo estimates were very similar to those estimating e_a_ from minimum temperature (−e_a_). However, when U and e_a_ data were missing, the slope was not so close to the unity ( *b* = 0.7345), and the intercept was not so close to zero ( *a* = 1.3922). In addition, due to *d* < 0.95, this combination indicated poor performance. The other combinations were unacceptable according to statistical indices and presented poor performance. As shown in Table 1, when RE is expressed as a percentage, these values range from 8.8% to 22.6% according to methods. The HS method showed the highest error at 22.6%, and the FAO56-PM method with missing U data showed the lowest error at 8.8% in estimating daily ETo. HS equation gave the highest MBE (0.550 mm d^−1^), whereas the FAO56-PM method with missing U data gave the lowest MBE (0.0009 mm d^−1^).

A study in a semi-arid highland environment by Benli et al. (2010) evaluated the performance of the FAO56-PM method for cases where RH, U, Rs, or all three parameters would be missing, comparing with weighing lysimeter measurements. They estimated Rs using Hargreaves’ radiation formula and estimated e_a_ accepting Tdew = Tmin. They used the average wind speed of U_2_ = 2 m s^−1^ instead of actual wind speed data because this value is the average wind speed of 2000 weather stations around the globe. The FAO56-PM with complete data ranked first in the study (RMSE = 1.32 mm d^−1^), FAO56-PM with missing U data ranked second (RMSE = 1.36 mm d^−1^), and FAO56-PM with missing e_a_ data ranked third (RMSE = 1.34 mm d^−1^). Similar to our research, the FAO56-PM method with missing U data estimated ETo accurately in their study. However, the HS equation (RMSE = 1.40 mm d^−1^; MAE = 54 mm) estimated ETo slightly better than the FAO56-PMT method (RMSE = 1.40 mm d^−1^; MAE = 55 mm) in their study, unlike our study.

A study in Southern Ontario, Canada, by Sentelhas, Gillespie & Santos (2010) evaluated the performance of FAO56-PM and alternative methods when data were missing. They used mean wind speed data observed in a close area. Using the procedure given in FAO56 Paper, they estimated Rs as a function of air temperature and also estimated vapor pressure deficit based on temperature data. The results proved that the FAO56-PM method estimated daily ETo accurately when wind speed and relative humidity data or all two parameters were missing. However, estimating Rs from temperature differences was not accurate enough in Southern Ontario because this approach resulted in poor ETo estimation. These results are in agreement with our study.

In our study, the FAO56-PMT method, using only a set of temperature data, could not accurately estimate daily ETo (Table 1). However, It provided more accurate results than HS and HS (A&G) equations. Similar results have been found in the studies performed by Trajkovic (2005) in Serbia, Popova, Kercheva & Pereira (2006) in the Trace plain area of south Bulgaria, Jabloun & Sahli (2008) in Tunisia, and López-Moreno, Hess & White (2009) in Pyrenees. However, Martinez & Thepadia (2010) reported that the HS equation produced more minor overestimation errors than the FAO56-PMT method. In their study, the FAO56-PMT method indicated the greatest errors in coastal areas, while the HS equation showed the greatest errors inland and island locations in Florida. Todorović, Karic & Pereira (2013) reported that where aridity prevails, the HS equation’s results were likely better than those of PMT. In contrast, PMT results were better for less arid climates, from semi-arid to humid. Martinez-Cob & Tejero-Juste (2004) determined that in windless locations of a semi-arid region in Spain, the HS equation gave unreliable estimates for daily ETo, and they recommended that the HS equation be used for 10-day periods. Torres, Walker & McKee (2011) reported that simple methods give less accurate results for daily ETo estimates than weekly and monthly ETo estimates.

### Estimating daily ETo using MLR models

Table 2 presents the MLR models developed for the thirteen combinations. The regression analysis (*t*-stat, *p*-value) was made to determine independent variables significantly affecting ETo at a 95% confidence level (Table 3). The values of the t-stat should be greater than 1.96 or less than −1.96 to verify the statistical goodness of the regression coefficients. The absolute values of the t-stat for all MLR models were greater than 1.96, except for Tmin in the MLR-2 model and Tmax in the MLR-9 model; the *p*-values confirmed this. The most significant variable in the MLR-4, MLR-8, and MLR-11 models is Rs, with the highest t-stat values. The most significant variable in the MLR-6, MLR-10, and MLR-13 models is Rs_n_, with the highest t-stat values. This indicates that Rs and n variables have more effect on estimating ETo than other variables in Adana Plain conditions. Similarly, Pereira et al. (2015) reported that Rs could dominate ETo estimation in mostly humid and sub-humid climates during summer, where the relative power of the vapor pressure deficit and wind term of the FAO56-PM is small compared to the radiation term. Several studies observed similar results to our research (Dimitriadou & Nikolakopoulos, 2022; Yirga, 2019; Perugu, Singam & Kamasani, 2013; Choi et al., 2018). In a study in Peloponnese, Greece, by Dimitriadou & Nikolakopoulos (2022), sunshine hours (n) and net radiation (Rn) significantly affected ETo more than the other variables. Among one-input models, the MLR model with n as the input variable exhibited the best performance (RMSE = 0.409 mm d^−1^, MAE = 0.320 mm d^−1^, *R*^2^ = 0.960). In contrast, the MLR model with U as the sole explanatory variable had the poorest performance in their research (RMSE = 2.001 mm d^−1^, MAE = 1.953 mm d^−1^, *R*^2^ = 0.030). In a study by Yirga (2019), for the Megecha catchment in Ethiopia to model ETo with MLR, the results of the multiple correlations showed that n was a strong positive correlation with ETo (*r* = 0.82), and the effect of n on ETo was more than other variables. Perugu, Singam & Kamasani (2013) performed a study in India, and the correlation analysis indicated that n, T, and U influenced the ETo more than other variables.

**Table 2:** Linear regression models.

**Model**	**Regression equation**
MLR-1	ETo =−0.149 + 0.117 × Tmax + 0.031 × Tmin
MLR-2	ETo =−1.581 + 0.157 × Tmax −0.003 × Tmin + 1.055 × U
MLR-3	ETo = 1.457 + 0.076 × Tmax + 0.070 × Tmin −0.017 × RH
MLR-4	ETo =−1.235 + 0.009 × Tmax + 0.075 × Tmin + 0.183 × Rs
MLR-5	ETo =−1.904 − 0.046 × Tmax + 0.162 × Tmin + 0.224 × Rs _T_
MLR-6	ETo =−1.110 + 0.024 × Tmax + 0.049 × Tmin + 0.166 × Rs _n_
MLR-7	ETo =−2.164 + 0.173 × Tmax −0.017 × Tmin + 1.108 × U + 0.005 × RH
MLR-8	ETo =−2.240 + 0.044 × Tmax + 0.048 × Tmin + 0.780 × U + 0.173 × Rs
MLR-9	ETo =−3.080 − 0.005 × Tmax + 0.127 × Tmin + 0.919 × U + 0.214 × Rs _T_
MLR-10	ETo =−2.100 + 0.059 × Tmax + 0.023 × Tmin + 0.771 × U + 0.156 × Rs _n_
MLR-11	ETo =−2.059 + 0.039 × Tmax + 0.053 × Tmin −0.002 × RH + 0.763 × U + 0.174 × Rs
MLR-12	ETo =−1.256 − 0.063 × Tmax + 0.181 × Tmin −0.018 × RH + 0.737 × U + 0.226 × Rs _T_
MLR-13	ETo =−1.227+ 0.035 × Tmax + 0.045 × Tmin −0.008 × RH + 0.687 × U + 0.158 × Rs _n_

**Table 3:** *t*-stat and *p* values of independent variables for MLR models.

**Model**	**Intercept**	**Independent variables**						
		**Tmax**	**Tmin**	**U**	**RH**	**Rs**	**Rs**_**T**_	**Rs**_**n**_
**MLR-1**								
*t*-stat	−1.331	21.174	5.773					
*p*-value	0.183	3.04 × 10^−93^	8.54 × 10^−09^					
**MLR-2**								
*t*-stat	−14.185	30.643	−0.588	28.639				
*p*-value	2.43 × 10^44^	4.3 × 10^−181^	0.556	8.1 × 10^−161^				
**MLR-3**								
*t*-stat	7.074	10.737	10.378		−9.227			
*p*-value	1.84 × 10^−12^	1.92 × 10^−26^	7.68 × 10^−25^		4.9 × 10^−20^			
**MLR-4**								
*t*-stat	−25.495	3.414	32.796			120.119		
*p*-value	1 × 10^−130^	0.0006	1.1 × 10^−203^			0		
**MLR-5**								
*t*-stat	−24.677	−10.665	40.731				66.337	
*p*-value	3 × 10^−123^	4.06 × 10^−26^	1.1 × 10^−292^				0	
**MLR-6**								
*t*-stat	−18.074	7.505	16.837					87.978
*p*-value	1.3 × 10^−69^	7.93 × 10^−14^	5.09 × 10^−61^					0
**MLR-7**								
*t*-stat	−9.432	23.570	−2.481	26.947	2.905			
*p*-value	7.49 × 10^−21^	2.1 × 10^−113^	0.013	2.5 × 10^−144^	0.004			
**MLR-8**								
*t*-stat	−66.160	26.151	32.121	69.480		178.820		
*p*-value	0	8.3 × 10^−137^	1.7 × 10^−196^	0		0		
**MLR-9**								
*t*-stat	−45.720	−1.318	38.918	42.866			79.509	
*p*-value	0	0.188	1.1 × 10^−271^	0			0	
**MLR-10**								
*t*-stat	−38.741	22.077	9.645	42.755				102.365
*p*-value	1.2 × 10^−269^	1.2 × 10^−100^	1.02 × 10^−21^	0				0
**MLR-11**								
*t*-stat	−29.728	16.698	24.754	60.724	−2.991	178.808		
*p*-value	1 × 10^−171^	4.35 × 10^−60^	6.1 × 10^−124^	0	0.003	0		
**MLR-12**								
*t*-stat	−9.782	−12.682	39.941	31.568	−16.431		84.232	
*p*-value	2.76 × 10^−22^	5.37 × 10^−36^	1.8 × 10^−283^	1.1 × 10^−190^	2.6 × 10^−58^		0	
**MLR-13**								
*t*-stat	−11.134	9.259	13.305	34.194	−9.064			103.871
*p*-value	2.82 × 10^−28^	3.68 × 10^−20^	2.34 × 10^−39^	9.7 × 10^−219^	2.13 × 10^−19^			0

RH data in the five models (MLR-3, MLR-7, MLR-11, MLR-12, and MLR-13) had less impact on the estimation of ETo with small *t*-stat values (Table 3). In a study in South Korea by Choi et al. (2018), Tmean, Tmin, Tmax, and n had strong positive correlations with ETo. In contrast, RH negatively correlated and had less effect on ETo, similar to our research. According to a study in China by Gong et al. (2006), FAO56-PM ETo was most sensitive to RH among the four climate variables, which vary by season and region and are affected by regional wind speed patterns, and followed this by Rs, T, and U. In the study by Nandagiri & Kovoor (2006), factor analysis indicated that U appears to be an essential variable in the arid climate. In contrast, n seems more dominant in subhumid and humid climates. Mohsin & Lone (2021) observed in the temperate Kashmir Valley for all the stations the strongest correlation of ETo with Tmin. In Egypt, Mattar & Alazba (2019) modeled ETo from climatic variables using GEP and MLR models. The results showed that using RH, U, or both in the models increased the performance of GEP and MLR models. Niaghi, Hassanijalilian & Shiri (2021) applied MLR models to Red River Valley with a subhumid climate to estimate ETo from climatic variables. They grouped input combinations into air temperature-based (Tmax, Tmin), mass-transfer-based (Tmax, Tmin, U), and radiation-based (Rs, Tmax, Tmin). Similar to our research, the radiation-based MLR model performed better than other combinations in their study (RMSE = 0. 68 mm d^−1^, MAE = 0.51 mm d^−1^, and R^2^ = 88%). In our study, as shown in Tables 4 and 5, the MLR-4 model (Tmax, Tmin, Rs) showed better performance than the MLR1 model (Tmax, Tmin) and MLR2 model (Tmax, Tmin, U).

**Table 4:** Performance statistics of MLR models in the calibration process.

**Model**	**RMSE** **(mm d^−1^)**	**MAE** **(mm d^−1^)**	**RE (%)**	**R** ^**2**^	**d**
MLR-1	0.915	0.750	22.5	0.3498	0.979
MLR-2	0.816	0.681	18.6	0.4822	0.985
MLR-3	0.903	0.749	22.3	0.3666	0.824
MLR-4	0.390	0.283	9.6	0.8818	0.980
MLR-5	0.594	0.444	14.6	0.7259	0.946
MLR-6	0.495	0.381	12.2	0.8096	0.965
MLR-7	0.815	0.679	20.1	0.4836	0.875
MLR-8	0.246	0.184	6.1	0.9528	0.999
MLR-9	0.474	0.342	11.7	0.8258	0.995
MLR-10	0.395	0.306	9.7	0.8787	0.997
MLR-11	0.246	0.183	6.1	0.9530	0.999
MLR-12	0.455	0.331	11.2	0.8394	0.995
MLR-13	0.390	0.302	9.6	0.8818	0.997

**Table 5:** Performance statistics of MLR models in the validation process.

**Model**	**RMSE** **(mm d^−1^)**	**MAE** **(mm d^−1^)**	**RE (%)**	**R** ^**2**^	**d**
MLR-1	1.117	0.940	24.2	0.4234	0.984
MLR-2	0.855	0.710	18.5	0.5826	0.992
MLR-3	1.097	0.930	23.8	0.4234	0.674
MLR-4	0.529	0.370	11.5	0.8860	0.954
MLR-5	0.881	0.710	19.1	0.7111	0.847
MLR-6	0.750	0.580	16.3	0.8479	0.894
MLR-7	0.851	0.700	18.4	0.5839	0.829
MLR-8	0.289	0.210	6.3	0.9514	0.999
MLR-9	0.581	0.420	12.6	0.8132	0.996
MLR-10	0.457	0.330	9.9	0.9084	0.998
MLR-11	0.288	0.210	6.2	0.9515	0.999
MLR-12	0.581	0.440	12.6	0.8225	0.996
MLR-13	0.463	0.340	10.0	0.9111	0.998

**Figure 3:**
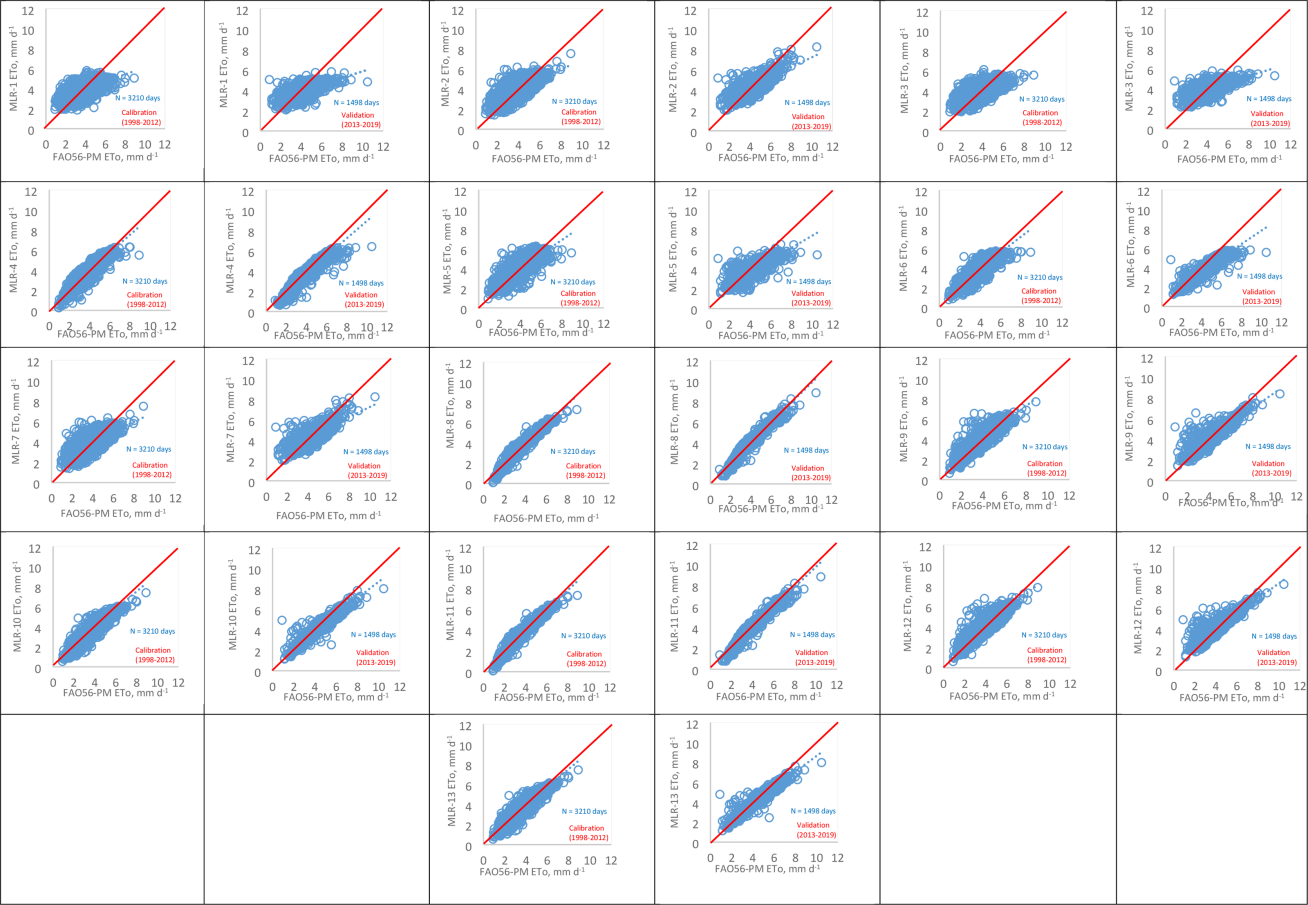
ETo values estimated by the FAO56-PM method against the MLR models for calibration and validation processes.

In Fig. 3, MLR model estimates were compared to the FAO56- PM method *via* scatter plots for the calibration and validation processes. The b (slope) values are close to one, and a (intercept) values are close to zero in MLR-8 and MLR-11 models with R^2^ of 0.9528, 0.9530 in the calibration process, and R^2^ of 0.9514, 0.9515 in the validation process, respectively. Also, MLR-4, MLR-10, and MLR-13 models performed well according to their b, a, and R^2^ values.

The values of RMSE, MAE, RE, R^2^, and d for MLR models during the calibration and validation process are presented in Tables 4 and 5. Among all models, the MLR1 model, estimating ETo with only Tmax and Tmin, showed the lowest performance with the values of RMSE of 0.915, 1.117 mm d^−1^, MAE of 0.750, 0.940 mm d^−1^, RE (%) of 22.5, 24.2, R^2^ of 0.3498, 0.4234 in the calibration and validation process, respectively. When Rs data were added to the MLR-1 model, the ETo prediction accuracy of the new model (MLR-4) increased by more than 52% for RMSE, MAE, and RE in the calibration and validation processes. In the MLR-6 model, Rs was estimated by n data using the Angstrom formula, and the MLR-6 model was designed with Tmax, Tmin, and Rs_n_. MLR-6 model estimated daily ETo with more than 32% accuracy than the MLR-1 model for RMSE, MAE, and RE values in the calibration and validation processes. Similarly, in a study by Yamaç (2021) in a semi-arid highland environment, when Rs data were added to the ANN1 model, which has Tmax and Tmin, the ETo estimation accuracy of the new model (ANN2) for MSE, RMSE, and MAE values increased by more than 30% in training and testing subsets.

In the MLR-5 model, Rs was estimated by Tmax and Tmin data using the Hargreaves radiation formula, and the MLR-5 model was designed with Tmax, Tmin, and Rs_T_. The results indicated that the MLR-5 model predicted daily ETo with more than 21% accuracy than the MLR-1 model, based on RMSE, MAE, RE, and R^2^ values in the calibration and validation processes. When RH data were added to the MLR-1 model, the ETo estimation accuracy of the new model (MLR-3) slightly improved with a range of 0.8% to 1.7% in the calibration and validation processes for RMSE, MAE, RE, and R^2^ values.

Only five models, MLR-4, MLR-8, MLR-10, MLR-11, and MLR-13, accurately predicted the daily ETo in the calibration and validation processes (Tables 4 and 5). The results showed that the MLR-11 model had the smallest RMSE (0.246−0.288mm d^−1^), MAE (0.183−0.210mm d^−1^), and RE values (6.1−6.2%) and the highest R^2^ (0.9530−0.9515) and d (0.999−0.999) for the calibration and validation processes, respectively. The statistical results of the MLR-8 model were nearly the same as the MLR-11 model (RMSE = 0.246−0.289 mm d^−1^; MAE = 0.184−0.210 mm d^−1^; RE = 6.1−6.3%; *R*^2^ = 0.9528−0.9514; *d* = 0.999−0.999). According to this, MLR-8 and MLR-11 models’ score was very good according to the criteria given by Stockle, Kjelgaard & Bellocchi (2004) for the calibration and validation processes. Therefore, the MLR-8 model is recommended in this study because it uses lesser climate data than the MLR-11 model. The MLR-8 model, which does not use RH data, can be successfully used to predict daily ETo in the study area. MLR-10 and MLR-13 models’ scores for the validation process according to mentioned criteria were very good, and the MLR-4 model’s score was good. Generally, the results of the models agree with the literature (Tabari et al., 2012; Dai et al., 2008; Yamaç, 2021), concluding that more climatic variables commonly increase model estimation accuracy. Tabari et al. (2012) evaluated MLR models’ different input combinations in a semi-arid highland environment in Iran. Similar to our study, the lowest performance was observed in the MLR model that only used Tmean (RMSE = 0.765 mm d^−1^, MAE = 0.621 mm d^−1^, *R*^2^ = 0.92) while the MLR model that used Tmean, Rs, RH, and U had the best performance (RMSE = 0.552 mm d^−1^, MAE = 0.442 mm d^−1^, *R*^2^ = 0.96) in their study. Dai et al. (2008) assessed the MLR models in northern China’s arid, semi-arid, and sub-humid areas. In the sub-humid area, the MLR models with four inputs (Tmean, RH, n, and U) and three inputs (Tmean, RH, n) gave similar results to the ANN model with the same inputs. MLR models predicted ETo accurately with RMSE = 0.202−0.273mm d^−1^, RE = 5.2−6.9%, *R*^2^ = 0.961−0.930, respectively. Yamaç (2021), in a semi-arid highland environment, evaluated ANN and kNN models. The results showed that more climatic variables increased the models’ predictive abilities, and the models with 6 inputs gave the best results in the study.

The main implication of our study is that MLR models developed for Adana Plain for estimating ETo have very satisfactory performance. The findings from our study are significant because agriculture accounts for 69% of global water withdrawals, mainly used for irrigation. This ratio can reach 95% in some developing countries (FAO, 2011). The FAO estimates that the world will need about 60% more food by 2050 and that irrigated food production will increase by more than 50% over the same period (FAO, 2017). To ensure sustainable development and water supply, particularly in arid environments, irrigation professionals need tools to estimate ETo at a large scale (Khoshravesh, Sefidkouhi & Valipour, 2017). Our study’s findings are significant for the agricultural managers and irrigation engineers in a region with similar climatic conditions to estimate ETo.

## Conclusions

One of the main goals of our study was to assess the FAO56-PM method using FAO56 procedures when climatic data were missing and evaluate HS and HS (A&G) equations. When wind speed data were missing, the use of the closest weather station’s average wind speed; when RH data were missing, the use of Tdew = Tmin; and when Rs data were missing, using the Angstrom formula for estimating Rs proved to be a great alternative to estimate ETo in Adana Plain. These approaches are strongly recommended for use in Adana Plain when missing climatic data. In contrast, Hargreaves’ radiation formula for estimating Rs did not perform well in estimating daily ETo. In addition, FAO56-PMT (a reduced set FAO56- PM), HS, and HS (A&G) equations could not estimate daily ETo accurately.

MLR was also employed to estimate ETo with different combinations of climatic variables in this study. Thirteen MLR models showed statistically significant results (*p* < 0.05). Rs and n were the most dominant climatic variables in estimating ETo in Adana Plain. These variables increased the models’ predictive abilities significantly. However, adding RH data to the models had a negligible effect on ETo estimates. The model using only Tmax and Tmin (MLR1) displayed the lowest performance. The models using Rs_T_ (MLR5, MLR9, and MLR12) did not perform well, while those using Rs_n_ (MLR10, MLR13, except MLR6) performed well in estimation daily ETo. The models using Rs (MLR4, MLR8, and MLR11) exhibited the best performance in estimating daily ETo. Especially the models using Rs and n data indicated a very satisfactory performance, so they may conveniently be applied in Adana Plain and other regions with similar climatic conditions with a reasonable degree of accuracy in the ETo estimation. Also, these results can guide irrigation engineers and agriculturists on which approaches and models will better predict ETo. So, it will help improve this region’s water resource management and irrigation scheduling. However, Our study’s limitation is that the MLR technique needs extensive data to avoid errors.

We recommend that the FAO56 procedures for estimating ETo when missing climate data should also be tested in the other region of Turkey. In addition, it is recommended that the MLR technique be studied in ETo modeling in other regions of Turkey. Since evapotranspiration is a complex and nonlinear phenomenon that depends on several climatic elements, future studies in this region should focus on artificial intelligence algorithms, and nonlinear models, such as MFP and MARS.

##  Supplemental Information

10.7717/peerj.15252/supp-1 Data S1 Raw data

## Additional Information and Declarations

### Data Availability

The following information was supplied regarding data availability: The raw data is available in the Supplemental File.
